# The Problem of Municipal Milk-Inspection1Paper read before the annual meeting of the Pennsylvania State Veterinary Medical Association, March 6, 1901.

**Published:** 1901-04

**Authors:** Jacob Helmer


THE JOURNAL

OF

COMPARATIVE MEDICINE AND

VETERINARY ARCHIVES.

VOL. XXII. *APRIL, 1901.* No. 4.

### THE PROBLEM OF MUNICIPAL MILK-INSPECTION.[1]

By JACOB HELMER, V.S.

FEW, if any, will deny the necessity of a more comprehensive system of milk-inspection in the towns and cities of this Commonwealth. The milk laws of Pennsylvania, upon which municipal legislation concerning milk is based, are, in the light of present knowledge and past experience, inadequate to the needs of the people. These organic laws deal mainly with the chemico-physiological side of the question, which determines the fat contents, and the amount of non-fatty solids and water.

In order to make more clear the ground covered by these laws, we will quote them in substance.

Pepper and Lewis' *Digest of Pennsylvania Laws*, p. 115, Section 45, treats of registration of dairies and milk depots in cities of the second class as follows:

"It shall be the duty of the Bureau of Health to make a complete registration of all dairies and milk depots in said cities, and to require the names of owners of different dairies or of persons dealing in milk to be placed upon each vehicle," etc. Then follows the penalty.

SEC. 46 forbids offering adulterated milk for sale, milk adulterated with water or any substance; also, milk from diseased cows and goats. Penalty follows.

SEC. 47 treats of the analysis of milk. Officers have the right to enter all places where milk is kept for sale to obtain samples for analysis.

[1] Paper read before the annual meeting of the Pennsylvania State Veterinary Medical Association, March 6, 1901.

SEC. 48. Milk wagons must be marked with the name of owner and locality.

SEC. 49 is devoted to the penalty for wrong marking.

SEC. 50 treats of adulterated and impure milk as follows: "The addition of water or of ice to milk is hereby declared an adulteration, and any milk obtained from animals fed on distillery waste or any substance in a state of putrefaction is hereby declared to be impure and unwholesome." Then follows the penalty for selling adulterated milk.

Page 415, Sec. 1, treats of adulteration with boric acid, salicylic acid, etc., or any other acid compound or drug.

Page 415, Sec. 2, deals with adulterated milk and cream.

Page 416, Sec. 3, declares that the enforcement of the law is imposed on the dairy and food commissioner. This section reads as follows:

"The agent of the Department of Agriculture, known as the Dairy and Food Commissioner, shall be charged with the enforcement of all the provisions of this act, and shall have all the power that is given him to enforce the provisions of the act by which he receives his appointment."—*Pennsylvania Laws*, p. 142, Sec. 2.

The State Live-stock Sanitary Board may take measures to protect supplies of milk from contamination. Page 377, Sec. 6, reads: "The Board and members thereof are hereby empowered to administer oaths or affirmations to the appraisers appointed under this act, that they may order and conduct such examinations into the condition of the live-stock of the States in relation to contagious diseases, including the milk supplies of cities, towns, boroughs, and villages, as may seem necessary and to take the proper measures to protect such milk supplies from contamination." —May 21, 1895, *Pennsylvania Laws*, page 91, Sec. 6.

State legislation relating to the sale of milk in the cities, towns, boroughs, and villages of Pennsylvania practically gives no authority to boards of health and councils for anything more than the appointment of an inspector to manipulate and analyze samples of milk. With few exceptions nothing more than this is done throughout the State. The milk laws of the State are good as far as they reach. Their deficiency consists in that they are not comprehensive enough.

But it seems quite natural that the law is as we find it. At the time the laws were enacted legislators had no warrant to pass laws that to-day may be rightly considered imperative. They did not know, and so they said we will secure natural milk for the people.

The idea that milk is a means for the conveyance of contagious and infectious diseases, not only such as may exist in the cow, but those existing in the human family; that milk not properly cured and handled may become a deadly poison to the infant that must consume it, and that the life-giving properties of the milk which render it so valuable as a diet depend for integrity or force upon the food, water, and hygienic surroundings of the cow. These are things in which our lawmakers had not been instructed, and naturally the evils did not occur to them.

Early in 1900 the economic section of the Green Ridge Woman's Club, of Scranton, undertook, among other reforms, to secure for the city an improved system of milk-inspection. The club had just finished dealing with the water supply of the north end of the city, and were justly elated with their success. Enthusiasm knew no bounds when the ladies began to grapple with the problem of the purity of the city's milk supply. Among the methods adopted by the club was the appointment of committees to visit milk depots, dairies, and dairy farms, and to report the conditions found; to have papers read before the club and in public; to collect the opinions of the leading physicians in the city on the question at issue; to secure the opinions and influence of some of the best and brightest among the laity and the influence of the press. It retained attorneys to consider the matter from every legal point of view; it corresponded with men holding positions in the dairy divisions of some of our universities; it corresponded with the secretaries of boards of health of various cities, and sought their advice. It debated all known forms of milk-inspection, and chose one that seemed best adapted to the needs of the city. Finally, as it naturally must, the matter in condensed form was laid before the board of health for criticism and action. In its deliberation assisted by counsel, the board discovered that parts of the code of milk laws submitted were illegal, but were assured such sections could be weeded out and legal substitution made.

Finally, in effect, the board decided that it or the councils had no jurisdiction; that under existing conditions it was an impracticable idea and would result in failure if for no other reason than that there was no law of the State which would permit either the passage of the law or its enforcement unless everyone connected with the matter worked in harmony and antagonism did not occur, which would inevitably defeat the proposed rules and regulations. Thus ended temporarily a splendid effort to gain a much needed municipal reform. If the views of the board which led to the abandonment of the proposed code of milk rules were not wholly true then the absence of an organic State law was used as a very convenient excuse. While you, as veterinarians, are familiar with the present methods of dairy practice and have more or less knowledge of dairy science, nevertheless it may be interesting to review the almost universal conditions which render it necessary to pass laws having sufficient scope to limit an evil about which the half has never been told.

The following few paragraphs are part of a report rendered by the visiting committee of the Green Ridge Woman's Club:

"At one 'depot' furnishing, the committee is glad to say, only forty quarts, the cans, not tightly covered, stood in a vegetable cellar twelve feet square, with no ray of light, the odor of which compelled hasty exit. A second depot furnished a vast amount of material for inspection. Bottling was done by hand. The cans and bottles were washed in a kitchen which was the living room of the family. The odor signified clearly that the one window in this kitchen—dining room, nursery, bottle washery—had never been opened. The proprietor earnestly denied ever scalding the brush, which cost twenty cents; also remarked that he kept his cans uncovered because milk would spoil otherwise. There was no ice. A third kept his cans of milk in the yard and peddled from a wagon. Five dairies visited gave the following results: first kept no ice; washed bottles with 'gold dust;' bottling room not clean; kerosene oil cans about; had used preservatives. Third showed nothing worthy of criticism; bottles and cans clean; scalding rags clean; shop also. Fourth—supply depot—ice plentiful, and cans kept in running water; commendable. Fifth, branch to above, same."

Another report of a different district stated: "In the first place visited the milk-house was clean and quite satisfactory, but the large barn, where thirty-four head of cattle were kept, was decidedly dirty, with a foul odor, showing that it had not been cleaned for some time. The next place inspected was a depot where large quantities of milk are received from the country. Here everything was quite clean, except it might have been suggested that the water in the tanks for cooling milk should be changed oftener, and no ice was kept, which was necessary during such warm weather as prevailed last week. One milk dealer merely rinses his cans and leaves the washing for the farmer to do some thirty-six hours or so later. How it is completed will have to be imagined. The dirtiest place visited was one where the stable is about as filthy inside and out as the imagination can make it. Dark and with no ventilation, we held our breath until we could get into the fresh air. Here six dirty, scrawny cows are kept with a plentiful supply of sour mash from the brewery. Coming out of the stable our eyes were met with a placard of diphtheria upon the house across the alley. The owner took us into the kitchen, which is also the living room, where he cares for and keeps his milk. The room is small and decidedly odorous. Here, too, we underwent the suspension of respiration as much as possible. The surroundings are composed of compactly built, filthy outhouses. It is a wonder there is not a wider spread of disease where said disease is sold for from five to eight cents a quart. Let every housekeeper make it her business to inspect personally the source of her milk supply."

In the report of another district visited the cleansing of bottles and cans was described as most undesirable. One proprietor disclaimed any accusation of ever having washed either cans or bottles, even in hot weather. "For," explained he, ingeniously if not very lucidly to the committee, "I do not want to be to blame for anything that happens, so I do not wash them at all, then I am not responsible at all, whatever," with which remarkable logic his listeners had to be content.

What matters it if the specific gravity of milk is right, shows the proper amount of butter fats and total solids, if it also contains dust and dirt from a filthy barn, particles of excrementitious matter from the cow, as well as the germs of a loathsome, and, perhaps, fatal disease?

The production of milk as an article of human food may well be given the first place of importance in the category of foods. In a pure state milk is a perfect food. It contains all the proximate principles to support human life. It will sustain life longer than any other kind of food taken alone. The child begins to grow upon milk, and much of its future health and strength depends upon the quality from a chemico-physiological and patho-bacteriological point of view. Aside from the child, milk is consumed by the adult. The invalid may subsist upon it. It has been estimated that the annual milk supply of the United States is nearly six billion gallons. In greater New York about 750,000 quarts are consumed daily.

In some cities large dairy companies turn the cans of milk fresh from the country over to the local milk-vendor or peddler, after first placing them in a row on the sidewalk. In order to secure the best can of milk for himself, the first vendor removes the cover, takes a swallow of the milk from perhaps all the cans along the line, and throws the remaining milk in his dipper, after each swallow, back into the can. The second does the same; and this continues until the vendors, may be a dozen or twenty men, have sampled all the remaining cans. The vileness of such a practice is enough, aside from the idea of the easy transmission of contagious diseases to the consumer of the milk.

The food inspector is engaged in a work of great consequence to the health of the community. His work is preventive of disease. It saves life. It fosters health. It gives security to the pleasure of eating. It is a work the practical importance of which is second to none by any member of our community. But since this work is so important, why is it not more thoroughly done than one man can do? Why is it not as necessary that it should be clean and free from dirt which may be so frequently found in the bottom of the milk pitcher where milk has not been properly strained off or filtered? Why should it be allowed to carry the germs of contagious and infectious diseases, such as typhoid fever, scarlet fever, anthrax, tuberculosis, and diphtheria? Why should it carry poisonous ptomaines, especially in the summer time, that kill more babies than all other diseases combined?

Dr. M. P. Ravenel, bacteriologist of the State Live-stock Sanitary Board, at the University of Pennsylvania in Philadelphia, who has had great experience with the bacteriological side of milk, asserts that a sample of the ordinary city milk is as pregnant with microbes as samples of city sewage that have been brought to him for examination.

Estimates have been made in a few other American cities. The number of bacteria in different cities varies widely. In the city of Boston in the spring it was found to have an average of about 2,300,000 per cubic centimetre. A cubic centimetre is about fifteen drops. The number of bacteria varied from 30,500 to 4,500,000. Milk should not contain more than 10,000 microbes per cubic centimetre. When it exceeds this number it is correspondingly dirty milk. The number found in the milk of a number of milkmen from Middleton, Conn., varied from 11,000 to 300,000 per cubic centimetre. Russell found 35,000 to 275,000 in April and 200,000 to 2,000,000 per cubic centimetre during May and June in the city of Madison, Wis.

To show what may be done to diminish the number of bacteria per cubic centimetre in milk through the use of systematic cleanliness and clean surroundings, I will read you a report for one week of the number of microörganisms found per cubic centimetre daily during the week beginning Saturday, January 6th, and closing Friday, January 12, 1900. This is by the Walker-Gordon Co., of Philadelphia:

| | | | |
|---|---|---|---|
| Saturday, | January | 6 | 2650 |
| Sunday, | " | 7 | 3250 |
| Monday, | " | 8 | 1625 |
| Tuesday, | " | 9 | 1525 |
| Wednesday, | " | 10 | 4600 |
| Thursday, | " | 11 | 4225 |
| Friday, | " | 12 | 875 |

These results were obtained from fresh cow's milk, which had not been either Pasteurized or sterilized. The examination was conducted at the Pepper Laboratory of Clinical Medicine, of the University of Pennsylvania.

The number of bacteria found varies according to the surroundings of the cows, the way they are prepared for it, and are milked; also upon the utensils, their cleanliness, the cleanliness of the attendants, and the way the milk is handled and shipped.

A competent inspection of the fountain-head of a city's milk supply—that is, the dairy—would result beneficially. It would cause the subject of dairy sanitation to be carefully studied. The State Live-stock Sanitary Board of this State and the Bureau of Animal Industry of the United States issue bulletins to the farmers on this and kindred subjects. No doubt much good results from this source of reading, but such reading is more incidental than systematic, because there is no incentive to reading and to the practising of new ideas except for curiosity.

In cities, producers of milk for sale, as a rule, read little or nothing. If they would they are not so apt to receive it from the government as the farmer is, because the latter is the owner of the dairy and the recognized source of the milk supply.

Milk inspection at the dairy would have the effect of stimulating inquiry as well as systematic study of the milk question, and the dissemination of knowledge which must result in the right production and proper handling of milk, which is the key to a pure and wholesome milk supply.

A milk inspector at the beginning of his work would find some cows crowded in dark stables, too small for the number of animals herded together, the walls and ceilings covered with dirt, dust, and cobwebs, the accumulation of years. There might be no drainage.

Cattle, especially on wet days, found standing above their ankles in mud and manure. Their bodies plastered with manure and dirt, especially in the winter time. Not only cows but other animals—horses, sheep, swine, and poultry—might also be found in the same apartment. The milk cans might be allowed to stand, with covers off, in a tub of water in the same room with the cows, absorbing gases, microbes and odors, without having itself been properly aerated. In some cases the milk is kept in cellars, where it is surrounded with various vegetables, decaying and otherwise, various kinds of meats, and everything that may be found in some people's cellars.

The successful milk inspector would practically start a school; for in places where milk-inspection is not in vogue few people understand how to handle this most susceptible article of diet. To implant new ideas while weeding out old ones, to inculcate cleanliness, to teach the relation of many species of bacteria to milk, would be some good resulting from his labors.

He would inspect every cow, the character of the food, and the water; the stable as to its area, the number of cubic feet of area space, the ventilation, drainage, the location and character of the milk house, the health and habits of those who handle the milk, the feeding, the bedding and cleaning of the cows, the manner of milking, straining, and cooling the milk. He would examine and inspect each cow in the herd. Animals with tuberculosis would be condemned; also those having cancers on the jaw or neck, enlargement of the udder, or any other physical condition which would contaminate the milk. He would pass no cow with any injury, disease, or lameness. He would require the walls and ceilings to be frequently swept and whitewashed, the floors and gutters to be cleaned before milking. The cow to be cleaned around the flanks and thigh as well as on the udder, in order to prevent dust and dirt from falling into the milk. These parts should be wiped off with a clean, damp cloth. Wetting the fingers while milking is unclean. The milker should wash his hands before beginning, and should put on a large, clean apron or a suit of outer garments used at no other time. The attendant upon a sick child or person having any contagious or infectious disease should neither milk nor have anything to do with the cows during the time the disease is at its height. If it is typhoid fever, great care should be exercised about the water in cleansing the milk utensils. If it is an infectious disease, like scarlet fever and diphtheria, everything should be done at the milk house, and no one exposed to the disease should handle the milk or have anything to do with it.

In intimate connection with this subject of the city's milk supply are vital statistics of the United States, exhibiting the number of deaths reported from each of the four diseases in persons under five years of age and in each year under five years:

| | Under 1 year. | 1 to 2. | 2 to 3. | 3 to 4. | 4 to 5. | Total. |
|---|---|---|---|---|---|---|
| Cholera infantum, | 20,263 | 5690 | 1139 | 281 | 124 | 27,397 |
| Convulsions, | 11,571 | 2008 | 796 | 277 | 417 | 15,009 |
| Diarrhœa, | 16,093 | 7023 | 2832 | 1236 | 236 | 27,950 |
| Debility and inanition, | | ...... | ...... | ...... | ...... | 20,679 |

Dr. Henry Baker, Secretary of the State Board of Michigan, comments upon these statistics as follows:

"My belief is that all the principal causes of infantile mortality are mostly results of ignorance. Ignorance of the practical application of bacteriology and mycology to every-day affairs, particularly to food, water, and food materials, on which human existence depends; ignorance of the effects of exposure, of that sort of knowledge which Herbert Spencer characterized as of 'most worth' —knowledge which tends directly to preserve life, knowledge which it should be the function of the public school system to make paramount, because the life of the people is the supreme law."

Taking up the causes of infantile mortality somewhat in the order of their importance:

*Convulsions.* One of the causes of greatest infant mortality is reported as "convulsions." Some of these are undoubtedly due to primary disease of the brain or nervous system; but it seems to be generally believed by those who have investigated the subject that the greater part of the deaths thus reported are really due to diarrhœa and other disease of the digestive tract. This is especially true of those who die of convulsions in or following hot weather.

Infant mortality from convulsions is, therefore, mostly due to ignorance of mothers, and others who care for infants, concerning the proper food for infants, and the proper guarding of that food from changes due to ferments, especially in hot weather.

*Cholera Infantum.* No one supposes that cholera infantum is wilfully caused by mothers or persons who have infants in charge, yet in some places the larger proportion of all children born die of that disease. That it results from causes which are preventable is shown by several facts: First, among the higher classes of people the mortality is very much less than among the less intelligent and less provident. Second, infants who are nourished wholly by mother's milk are almost wholly exempt from cholera infantum. Third, the disease is undoubtedly caused by changes in the infant's food or drink, due to bacteria, fungi, or some sort of micro-organism.

But cows are also victims of a number of acute maladies. Scarcely a herd exists that does not contain a milk-producing cow that is not affected one way or another. Occasionally a herd is a hot bed of disease.

Again, typhoid fever bacilli have been found in milk. Hart reported fifty epidemics of typhoid fever with 3500 cases, and Dr. Fruman, of New York, collected records of fifty-three epidemics with 3226 cases. In all of these cases the typhoid bacilli were distributed by milk infected with that germ. Diphtheria is another disease transmitted by means of infected milk. Dr. Hart collected statistics of seven epidemics of diphtheria with 500 cases. Dr. Freeman, of New York, obtained a record of eleven epidemics with 501 cases. Of these, eighteen epidemics were transmitted by means of infected milk. In Buffalo on September 4, 1894, nineteen cases of typhoid fever were traced and their origin discovered on a dairy farm. In August, 1895, the health department traced eighteen cases, and found their origin on a farm furnishing milk to the city. Again, on March 9, 1896, fourteen cases were traced to the country. Each of these three outbreaks were closely associated with the routes of milk vendors. In the light of such reports common-sense shows that outbreaks of contagious diseases communicated through milk as a medium are prevalent throughout the civilized world wherever milk is consumed in the raw state.

Some observers who have devoted study to the question of controlling the purity of milk and milk products hold that it is not logical to make an artificial standard to which all must come, but to allow the seller to guarantee his own product. Strictly arbitrary systems in which compulsion is the rule cannot eventually succeed, not only because of the antagonisms they arouse, but because of the impracticability of suddenly lifting the dairy business from its common every-day habit and practice to a plane more in accordance with dairy science. No system can be absolutely perfect or entirely successful. The question is not as to the most perfect system, but the most practical; the one which, all things considered, will yield the best results both in protecting the consumer and in giving an impetus to better methods of dairy practice.

Cities successfully working along lines parallel with the artificial standard system do not command dairymen and individual milk sellers to conform to a set of arbitrary rules, but make the sale in the city of milk from every farm depend upon a proper and reasonable condition, viz., that if the seller does not conform to the rules and regulations of the city the latter will not grant a license for said product to be sold within her borders. This is quite a different proposition. To my knowledge this principle has not failed in any place where it has been adopted and tried. The difficulty is to get the system started. But it is better to begin late and begin right than to start wrong.

The other theory, to let each producer guarantee his own product, is apologetic and is based upon reward of merit. In accordance with this system a board of health would say to milk vendors and owners of milk depots on condition that you will guarantee to have your herds inspected and to keep your milk up to a high standard and conform to all the rules and regulations of this city, etc., we, the board of health, agree to issue to you a certificate of merit. In order that said certificate may be of value to its possessor, the board of health agrees to have made a weekly test of the milk furnished by the holder of the certificate, and furnish weekly publications in the press above the recommendation of the board. The vendor also agrees to be subjected to an adverse report should his product at any time fall below the standard prescribed, said adverse report to continue indefinitely or as long as the vendor shall fail to prove the milk to again merit favorable consideration. This method antagonizes no one. It is beautiful. It is easy. It costs nothing to start it, nothing to end it. The competition for a paltry newspaper "ad" is expected to continue until all producers and vendors are in the fold. But we are not told how long this milk millenium will continue.

Is not the system best in which education is linked with reasonable compulsion and all backed by the strong arm of the law? The State laws governing the milk traffic in Pennsylvania place the power for their enforcement into the hands of the dairy and food commissioner. This is of little practical value to the people.

Apart from the duties of the dairy and food commissioner each town, city, and borough should have power from the State to make laws broad enough to properly regulate the milk traffic and to enforce them when made.

Although the jurisdiction of a city is confined to the city limits, in a question of this nature its influence extends beyond. In

Minneapolis and other places it extends as far as their markets, embracing other counties.

The sale of milk in any city should be made contingent upon the possession of a license.

The rules and regulations of cities and towns governing milk-inspection—and scarcely any place is without them—should include laws for the thorough and systematic inspection of dairies and dairy herds.

The law should provide for this purpose a corps of competent inspectors. These inspectors should inspect all places where milk is produced, dairies and dairy farms as well as places where it is kept for sale, as stores, wagons, depots, etc.

The rules and regulations should be reasonable and made possible to be complied with, but in no case should a license be issued by the authorities, the board of health, or councils, or both unless the conditions have been fulfilled. Such facts would be determined from the inspection report—a form provided for this purpose and submitted over his signature. On any subsequent tour of inspection if the conditions on which the license was granted were no longer observed the latter would be revoked.

Many cities, including Scranton, have laws in the health manual which cannot be enforced. Concerning milk, among other things it reads "that he (the milk inspector) shall require all dealers, wholesale and retail, to register with the Secretary of the Board of Health and take out license for one year."

The form reads as follows: "I, Mr. , of the city of Scranton, desire to register as a retail milk dealer and hereby agree, if a license is granted me, to conform to all the rules and regulations prescribed by the Board of Health governing the production and sale of milk. My place of business is at No. ."

The matter is wholly left to the milk inspector; the requirements for license are not even printed, and appear to be granted on the general appearance of the seller's outfit. The Board of Health have issued a number of requests asking the farmers to have their herds examined with tuberculin.

The proposition that boards of health through councils have power to enact laws extending beyond State provisions, providing the enactments do not conflict with the State laws, is fairly answered by the counter proposition that, especially in larger places, it is difficult to harmonize antagonistic elements when seeking to enact laws on assumed authority.

A State law empowering councils of cities to enact legislation providing for the inspection of dairies and dairy herds is the key-note of the problem of municipal milk-inspection. It is a paramount consideration. Other elements in the problem, such as the position of the laity, the taxpayer, the farmer, boards of health and councils are educational questions. The laity always desire the best. In view of the benefits to be obtained it would impose no additional burden to which the taxpayer could object.

The producer in the country is no less interested than the consumer in the city, and has not been known to obstruct a reasonable measure of reform, especially where it has been possible to comply with the same. The producer would lay most stress on questions of fairness, and that all of his class must receive one and the same treatment. The chances are favorable that his product could command a higher price in the market. If some left the dairy business, others would be that much better off. The question of not being able to afford additional expense on account of the cheapness of milk would bring up the question of supply and demand in relation to the price of milk.

Bureaus would occupy an anomalous position when not providing for the enforcement of existing laws for the health of their cities. Councils would be governed by their health bureaus. These in turn would be sustained by the press and the people. The only great danger would arise from political influences, underhand methods, men offering their services to fight a project for a money consideration.

In April, 1895, the Legislature of the State of Minnesota passed an act relating to the inspection of milk and of dairies and dairy herds, and to provide for the licensing and regulation of the sale of milk in cities. The law reads as follows :

*Be it enacted by the Legislature of the State of Minnesota :*

SECTION 1. The City Council of any city may be ordinanced to provide for the inspection of milk and of dairies, and of dairy herds kept for the production of milk within its limits, and issue licenses for which no fee shall be charged for the sale of milk within its limits and regulate the same, and may authorize and empower the Board of Health to enforce all laws and ordinances relating to the production and sale of milk and the inspection of dairies and dairy herds producing milk for sale or consumption within such city, and to appoint such inspectors, experts, and chemists as are necessary for the proper enforcement of such laws and ordinances, their compensation to be fixed by the City Council, and such inspectors, experts, and chemists shall be possessed of such necessary powers within the limits of such city as shall be prescribed by ordinance, but no such ordinance shall conflict with any law of this State.

SEC. 2. Nothing in this act contained shall affect or interfere with any of the powers and duties conferred upon the State Dairy and Food Commissioner or his deputies and agents by any law of this State.

SEC. 3. This act shall take effect and be in force from and after its passage.

Approved April 26, 1895.

In the following June the Councils of the city of Minneapolis passed its splendid code of rules and regulations governing the milk traffic of that city. The code was adopted on June 21st, and has been in successful operation ever since.

Why cannot additional legislation in the form of a concise law governing the inspection of dairy and dairy herds be secured on the statute books of the Keystone as well as in other States?

In conclusion, if to obtain such additional legislation is considered by you a necessity to advance the cause of municipal milk-inspection, is there any better time than the present to take some preliminary steps to secure it?

DISCUSSION.

Dr. Marshall: Mr. President: The paper we heard this morning by Dr. Helmer was so carefully prepared that there is very little more to be said on the subject of municipal milk-inspection. The ground I intended to cover this afternoon was to tell you what has been done voluntarily. I have been connected with dairies working along this line for some time, and know something of how they are managed. There are a few dairies in this city which are trying to conduct business on thoroughly scientific principles. In most instances, however, milk inspection is merely done on the platforms, and the Pediatric Society of Philadelphia, a class of physicians who devote most of their time and attention to the treatment of children, and who are trying to get a wholesome milk, have arrived at the conclusion that this could only be done in some voluntary way at present. They established a commission about a year ago to outline the requirements of a scientific milk inspection, and I will read you the conditions from their report.

1. There shall be a Milk Commission of the Philadelphia Pediatric Society, whose duty it shall be to have examined milk submitted to them by dairymen and certify as to the result of such examination.

2. The actions of the Commission shall be reported from time to time to the Society and shall be subject to its approval.

3. The Commission shall consist of four members beside the President of the Society, who shall be a member *ex-officio*. The members shall be appointed yearly by the President as soon as possible after his election. The Commission shall elect a chairman and a secretary from their number.

4. No statement for publication or information to any dairyman shall be given by or in the name of any individual member, but only after consideration by the Commission and in the name of "The Milk Commission of the Philadelphia Pediatric Society.

5. The Commission will hold itself in readiness to examine milk from dairies desiring this examination, and to certify to the good quality of milk which comes up to the standards fixed by it. It is understood that only the milk of dairies, and not that of milkmen who merely serve milk bought by them, will be examined by the Commission.

6. The method of examination and certification to which the dairyman or his agent shall agree to submit shall be as follows:

7. The Commission shall select a bacteriologist, a chemist, and a veterinary inspector. The bacteriologist shall procure a specimen of milk from the dairy, or, preferably, from delivery wagons, at intervals to be arranged between the Commission and the dairy, but in no case at a longer interval than one month. The exact time of the procuring shall be without previous notice to the dairy. He shall test this milk for the number and nature of bacteria present in it, to the extent which the needs of safe milk demand. He shall also make a microscopical examination of the milk for pus-cells. Milk free from pus and injurious germs and having not more than 10,000 germs of any kind or kinds to the cubic centimetre, shall be considered to be up to the required standard of purity.

8. The chemist shall in a similar manner procure and examine the milk for the percentages of proteids, fat, sugar, mineral matter, and water present. He shall also test its chemical reaction and specific gravity, and shall examine it for the presence of foreign coloring or other matters or chemicals added as preservatives. Standard milk shall range from 1.029 to 1.034 specific gravity, be neutral or very faintly acid in reaction, contain not less than from 3.5 per cent. to 4.5 per cent. proteid; from 4 per cent. to 5 per cent. sugar, and not less than 3.5 per cent. to 4.5 per cent. fat, and shall be free from all contaminating foreign matter and from all addition of chemical substances or coloring matters. Richness of cream in fat shall be specified and shall vary not more than 1 per cent. above or below the figure named in selling. Neither milk nor cream shall have been subjected to heat before the examination has been made, nor at any time unless so announced to the consumer.

9. The veterinary inspector shall, at intervals equal to those of the bacteriologist and chemist, and without previous warning to the dairy, inspect the cleanliness of the dairy in general, the care and cleanliness observed in milking, the care of the various utensils employed, the nature and quality of the food used, and all other matters of a hygienic nature bearing upon the health of the cows and the cleanliness of the milk, including also as far as possible the inquiry into the health of the employés on their farms. He shall also see that the cows are free from tuberculosis or other disease.

10. The charges made by the experts shall be—for the veterinarian $10, and $5 for each of the others for each examination; this amount to be paid by the dairy at the time of the examination and without regard to whether the report is favorable or unfavorable. The experts shall make their examinations when, and only when, notified to do so by the Commission. Any dairy the milk of which shall be found by the examiners to be up to the standard of the Commission shall receive a certificate from the Commission, which shall read as follows:

*Milk Commission of the Philadelphia Pediatric Society.*

Date

The Veterinary Inspector of the Commission has examined the dairy of Mr. and reports it to be well kept and clean, and the cows to be in a healthy condition.

The Bacteriologist reports that the milk does not contain germs beyond the limits of the standards of the Commission.

The Chemist reports that the milk is of standard richness, and that he has discovered in it no impurities, coloring matters, chemical preservatives, or harmful substances.

The Commission certifies to these statements of the examiners. It is understood and agreed to by the said Mr. that this certificate is good for not more than from date, when another examination is to be made.

Signed by the Commission.

11. In case an examination shows the milk not up to the standard the dairy may have a re-examination made within a week or within a short time, at the discretion of the Commission.

12. The Commission for 1900 have selected –

As Veterinary Inspector, Dr. C. J. Marshall.
As Bacteriologist, Dr. M. P. Ravenel.
As Chemist, Dr. Henry Leffmann.

13. Milk furnished by the dealers to whom certificates have been issued shall be furnished to consumers in glass bottles hermetically sealed in a manner satisfactory to the Commission. In addition to the sealing, and as a guarantee to the consumer that the examination has been regularly conducted, there shall be pasted over the mouth of the jar, or handed to the consumer with every jar, according to the discretion of the Commission, a certificate slip which shall read as follows:

*Philadelphia Pediatric Society, Milk Commission Certificate.*

Milk from the dairy of Mr. has been recently examined by the experts of the Milk Commission and found to be fully up to the required standards. Another examination is to be made within a month, and, if satisfactory, new labels for the bottles will be issued, dated .

Notice the dates.

At the present time there are three different milk dealers handling milk under these conditions. There is no objection to any milk dealer or farmer complying with these conditions, and furnishing the milk to the Society and getting their recommendation. The great objection to this work is the price of the milk. Five farms I have investigated, and asked them about the price for which they could furnish milk under these conditions. None of these can make it under five cents a quart, figuring on the interest of the money invested, on cows, barns, help, and all these things—it costs five cents per quart to make the kind of milk. They all sell this milk for ten cents per quart in quantities of more than two quarts, or twelve cents per single quart. The smallest farm is furnishing milk from seventeen cows, having it certified, and most of it is required. Another farm has one hundred and eighty cows, and most of the milk goes to New York City, and is sold for fifteen cents per single quart and twelve cents where several quarts are taken. There is another farm near this city which did not send in for certificates that is furnishing just as good milk whose milk is higher in fats—about 6 per cent.—and this farmer is not going to send in a certificate that his milk is 3½ to 4½ per cent. fats (which is what the Commission requires) when it is running 6 per cent. This difficulty could be gotten over by adding skimmed milk and diluting down to 4½ per cent. He says he can sell all his milk, and he has milk from over one hundred cows, and not have the recognition of the Pediatric Society. He has not as yet come into the Commission, but we are in hopes he will. I think this Society has one of the best methods I have ever heard of. It furnishes milk in an ideal way and should be more universally adopted. The only objection is the price. I know one farmer who was selling milk at the regular wholesale price and attempted to produce certified milk to sell in this city. He had a farm of about eighty acres, and was out of debt, and made money on his farm under the old system, and he and one son did all the work; but when he attempted to run things in the new way he had to go into debt for $2000, spent all his time in the dairy—he, his son, and another man—and had no time to leave the farm. It kept them busy from 4 o'clock in the morning until 10 o'clock at night, and cost him five cents per quart to make the milk and he sold it for four. He got disgusted and quit. I think any intelligent farmer can follow out all these requirements and will get below 10,000 germs per c.c., and there seems to be a demand for this kind of milk, and while the number of people requiring it is limited at present, I think it will increase. There are two dealers here in the city—Wills and Supplee—who have attached this to their regular milk route, and it costs them nothing extra to deliver certified milk. They get their milk certified to, and are ready to furnish milk as there is demand for it, and will keep as many cows as are necessary to produce that kind of milk. Their business is growing a little. They get milk from seventeen cows at present, which supplies their demand. Mr. Abbott sells milk from the Thorndale dairies, obtained from fifty cows, milk that is certified to, and he also sells milk that is not certified, which goes into the ordinary milk supply. This Pediatric Society does not require the tuberculin test —they leave that to the veterinarian. If he thinks any animals ought to be tested with tuberculin, he has the right to test them. At the present time there are no dairies furnishing this certified milk that are not having their cattle tested with tuberculin. This is the only way they can carry the work out properly. The report I have to fill out each month reads as follows:

*Milk Commission, Philadelphia Pediatric Society; Report of Veterinarian.*

Philadelphia, , 190 .

Secretary of the Milk Commission,

Dear Sir: I have examined, as requested by the Commission, the dairy of at and find the following conditions:

1. milking cows; hospital cows; dry cows; cows added since last report; cows now in quarantine; cows recently calved; cows sick since last report. Total number of cows in herd of which have been tested with tuberculin.
2. Condition of milch cows at present, good.
3. Food employed.
4. Condition of stables: ventilation, ; heat, ; floors, ; troughs, ; cleanliness, etc., ; dimensions of stables, ; condition of other buildings good.
5. Health of employés and their families, as far as ascertained, is good.
6. Source and character of water in dairy and stables.
7. The general precautions of cleanliness in milking and the care of the milk are satisfactory.

I, therefore, recommend that milk from this dairy be submitted to the Bacteriologist and Chemist of the Commission for their examinations.

Yours truly,

(Signed) C. J. MARSHALL, V.M.D.,
Veterinarian.

In making these examinations I do not go through the herd and make a physical examination. I ask the superintendent as to their condition. Most of these dairies have a superintendent who is a graduate of an agricultural college. If any of the cows appear sick, he reports to me, and I go through the herd. I look especially to see if the cows and stables are kept clean, whether the water supply and ventilation are all right, the condition of the bottles and milk rooms, that the men are putting on clean suits of clothes, and all that is required by the Pediatric Society is fulfilled. I find where they are trying to furnish this kind of milk they are only too glad to follow any suggestions I may make that are of benefit to them.

The following are the forms used by the Chemist and Bacteriologist:

Philadelphia, , 190 .

Secretary of the Commission,

Dear Sir: At the request of the Commission, received on , 190 , milk from the dairy of labelled was obtained by me on , 190 , at Street, at o'clock m., and examined at o'clock m., with the following results:

Total solids (by evaporation of weighed amount in platinum basin), ; fat (Leffmann-Beam method), ; ash (by burning off solids obtained as above), ; total proteids (Kjeldahl-Gunning method, factor 6.25), ; preservatives and added color, none; specific gravity, .

I, therefore, recommend the milk as coming up to the standards adopted by the Commission.

I find the milk bottles to be sealed in the manner prescribed by the Commission. Yours truly,

(Signed) Dr. Henry Leffmann,
Chemist.

Philadelphia, , 190 .

Secretary of the Commission,

Dear Sir: At the request of the Commission, received on , 190 , milk from the dairy of labelled was obtained by me on , 190 , at Street, at o'clock m., and examined at o'clock m., with the following results:

Number of bacteria per c.c. of milk .

I have been unable to detect any pathogenic organisms or evidence of purulent inflammation of the udder.

I, therefore, recommend the milk as coming up to the bacteriological standards adopted by the Commission.

I find the milk bottles to be sealed in the manner prescribed by the Commission. Yours truly,

(Signed) M. P. Ravenel,
Bacteriologist.

Dr. Lowe, President of the New Jersey State Veterinary Association, said: I appreciate the importance of the bacteriological and chemical investigation, and its application to the analysis of milk, as much, probably, as any gentleman here, but I think that the important thing to do is to begin with the production of the milk, and the inspection should be begun in connection with the production of the milk—that is, in the first place, in the examination of the condition of the stables, hygienic condition of the stables and the animals themselves; the food of these animals, and the water supply of these animals. I know that you cannot produce good milk unless you have good, sound, healthy animals to begin with, and unless they are fed on good, wholesome food. That is the principle involved in the first place. He called attention to the importance to-day of the veterinarian on boards of health; the relation of veterinary medicine to public health, and the ignorance of many otherwise educated men, including M.D.'s, upon this subject. He added: Do not begin by asking for legislation on this subject, but begin by getting the dairymen, stockmen, and consumers to appreciate the value of the work you contemplate, and when the public recognizes the value of this work that the veterinarians are doing it is very easy to obtain legislation, for when the public need a thing the legislators will give it to them, but they are very slow to make a law unless the public are ready to receive it.

Dr. Ranck: There are just a few points I would like to bring before the members. They have already been gone over. There are several tests for milk to determine the cleanliness of the milk before it comes to the consumer, so you can determine definitely how the milk was handled. For instance, in the acidity of milk, or milk that is carelessly handled. The acidity of the milk is one of the most reliable tests to determine the character of the cleanliness of the milk. Also in regard to the bacteria. Of course, when this question of bacteria comes before us—bacteria in milk—it must be definitely understood that all bacteria in milk are not harmful. It is very necessary for bacteria to be in milk. It is very necessary, for instance, in the production of cheese and all the by-products of milk. Of course, these germs are floating about in the atmosphere continuously. There are harmful bacteria, and also harmless ones that are necessary in milk. The Pasteurization and sterilization, adopted by many dairymen, are detrimental to milk. Men who have made investigations in this matter report that Pasteurization and sterilization of milk do positive harm. It is only the bacteria that are productive of disease that are harmful. The stable hygiene is one of the principal things we will have to look forward to. The veterinarian should take the field that the chemists are now in. The chemist has no right to be a milk inspector. The veterinarian should be equipped so that he is fit to inspect milk. What does the chemist know in regard to the physiological condition of milk? Even the physiologists know very little, and why should we depend upon the chemist who has never studied physiology? How does he know how to test milk in a competent manner? We, as veterinarians, should be able to test the milk from one end of it to the other.

Dr. Michener: I have nothing to say about this paper except that it was a well gotten-up paper and beautifully rendered. My opinion is that the paper would do a great deal of good if put into pamphlet form and spread broadcast over the State and read before each farm club. I congratulate the gentleman who read the paper as being one of high intelligence.

Dr. Morris (M.D. and large cattle-owner) being called by the President, responded as follows: I hope you will excuse me. I do not know that I have anything new to say. I said a year ago at a meeting of the Keystone Veterinary Association, when Dr. Marshall was there and introduced the subject, that I was delighted to find that the Pediatric Society is working now at what I began to work at thirty years ago, and I was very much in hopes that they would succeed in their work. I am very glad that there are five—certainly three—milk producers in the neighborhood of Philadelphia who are ready to meet the conditions of that Society and supply milk that is satisfactory to the city. So far good. I think there has been a decided gain in the thing being done, but, on the other hand, there are a great many things which we have to take into consideration besides that. The quantity of milk that is thus supplied in proportion to the whole amount demanded for the nutrition of the infants of Philadelphia, is only as a drop in the bucket. Imperfect as our milk-inspection may be—and I do not regard it as absolutely perfect—still it is infinitely better than we had before, and, as far as I know, better than that which prevails in any other city on this continent. I think it is honestly and well carried out, that the general character of the milk furnished the community is very much better than it was twenty-five or thirty years ago. At that time I drew up a series of tables showing the existing mortality in children under five years of age from poor milk supply, and I showed that in Philadelphia alone we were losing from twelve to fourteen hundred children a year under five years of age who might be saved by an improved milk supply. In New York a rigid inspection was established which threw out about 200,000 quarts of watered milk daily for a while; and that year the deaths from cholera infantum, etc., fell from 17,000 to less than 14,500. A comparison of the population of New York and Philadelphia then showed that we were losing 1200 children a year by improper milk supply. This shows how far the whole community can be affected. Suppose a proposition was made that by improper feeding we should annually kill off the population of Girard College; suppose that way should be taken to get rid of the pupils of Girard College. There are twelve to fourteen hundred orphans there dependent upon charity. When we do not have sufficient milk supply in Philadelphia, and do not take care to make our milk supply what it should be, we are virtually doing that thing in Philadelphia—that is, the price we are paying here in Philadelphia for imperfect, poor milk supply.

Now, efforts have been made by these gentlemen—and a very praiseworthy thing it is—to secure an excellent quality of milk, and a certified quality, so that persons that are willing and able to pay for the better article can do it. How about those that cannot? The trouble, as far as I can see it with the proposition of the Pediatric Society, is the amount of expense entailed. Dr. Marshall said very properly that milk cannot be produced under healthy conditions for less than five cents per quart. Our milk men are in the habit of getting two and one-half and three cents per quart, that is all. How can they afford to do it? The additional expense which is involved in such proper examination and registration is so great that the milkmen cannot undertake to do anything of the sort. It is just absolutely out of the question, and, as one of the gentlemen has said to-day, the idea is to look out for the health of the community at large. It is not only for those who have large incomes—incomes of $20,000 a year—who would pay twenty cents a quart for milk if necessary, but for the thousands and thousands of laborers and their families to whom a difference of one cent a quart in milk is a very important consideration. That is another side of this question. It has so many sides we hardly know which one to take first. Suppose a farmer has 400 cows that are yielding milk, and he is sending 1800 to 2000 quarts daily to the city, he can afford to have an inspection regularly. The whole amount of that inspection is about twenty dollars a month, from Dr. Marshall's paper. Suppose he has that twenty dollars a month—these 2000 cents—if he is sending 50,000 quarts or 60,000 quarts, the twenty dollars for inspection is a small thing. That is not the case generally. Nine-tenths of the men are not milking over from ten to twelve cows. If you add twenty dollars a month to their expense it will be utterly impossible for them to sell that milk. It adds so much to the price of milk that it is a prohibitive thing to have, and I would suggest to that

Society some such modification of their mode of inspection and certification that a large number of the small producers could very well be induced to have their milk properly inspected, registered, and certified. That is the first thing that occurred to me while I was listening. Another was in regard to the character of the milk which is supplied. When they get down to the percentage of 3½ and 4 per cent. of fats, whereas all our best cattle are making 5½ to 6 per cent. of fatty material, there is an opportunity open for the adulteration of that milk or its reduction, so as to bring it within the standard adopted by the Society, and I do not think that would be an advantageous thing for the consumer at all, because the character of the milk is more or less changed, and its adaptability is not as good in that mixed and modified milk as it is in the secretion that comes from the cow. This I speak of from experience. I am also pleased with what has been said as to the Pasteurization and sterilization of milk ; that also, I am satisfied, is a mistake. We do not want that milk. We want good, whole milk as it comes from the cow, at the least rate that can be made remunerative to the farmer. If you put it at such a price as is unremunerative to the farmer you tempt that farmer to adulteration.

What is the absolute cost to the farmer of milk? With hay at fifteen dollars per ton—and I have gone over these figures very carefully—milk costs the farmer four cents per quart to produce from ordinary cattle, and he is making no money on it at that. Milk costs to deliver in the city, over and above the cost to the farmer, one-half cent a quart for freight, and three cents a quart for the delivery, so that when milk is sold or delivered on the street—I am speaking of milk at less than seven and a half cents per quart—it is sold at a loss to somebody, and, therefore, it is not being sold to the best advantage of the community. Registered milk, from the fact that it involves a larger amount of investment in blooded animals, and the taking care of them, cannot be sold at less than ten cents without loss. Now, then, compare those figures with the income of the average workman or mechanic, and you will see that he cannot pay more than that amount for his milk. Nor am I drawing these figures only from our experience here in this section. I went over a number of tables showing the price at which milk is sold in France, Belgium, Holland, Great Britain, in New England, and in the West, and I found that, after converting the various currencies into our own, the milk, good milk, was four cents per quart. So there does seem as if there were some law of economy which you may regard as a fixed fact, and from which we may argue as to what can be done for the benefit of the community in producing a good, wholesome article of food. I hope this Society will take it up and do what can be done. It is the all-important thing—the healthy character of the animal from which this is produced ; healthy surroundings ; health of the attendants and distributors of the milk are of the first importance to the community at large, and I feel as if that were a work in which we could engage to the very great profit of the community as well as honor to ourselves.

Dr. Jacob Helmer: I will say that a bill on the subject is at this time pending in the Legislature. I have tried to get a copy of the bill, but have been unable to.

The President: Dr. Pearson is not here now. Probably you had better bring that up at a later time for the Board of Trustees to incorporate in their report. Is that satisfactory? (Dr. Helmer nodded assent.)

